# Phytogenic Products and Phytochemicals as a Candidate Strategy to Improve Tolerance to Coronavirus

**DOI:** 10.3389/fvets.2020.573159

**Published:** 2020-10-20

**Authors:** Youssef A. Attia, Mahmoud M. Alagawany, Mayada R. Farag, Fatmah M. Alkhatib, Asmaa F. Khafaga, Abdel-Moneim Eid Abdel-Moneim, Khalid A. Asiry, Noura M. Mesalam, Manal E. Shafi, Mohammed A. Al-Harthi, Mohamed E. Abd El-Hack

**Affiliations:** ^1^Agriculture Department, Faculty of Environmental Sciences, King Abdulaziz University, Jeddah, Saudi Arabia; ^2^The Strategic Center to Kingdom Vision Realization, King Abdulaziz University, Jeddah, Saudi Arabia; ^3^Animal and Poultry Production Department, Faculty of Agriculture, Damanhour University, Damanhour, Egypt; ^4^Department of Poultry, Faculty of Agriculture, Zagazig University, Zagazig, Egypt; ^5^Forensic Medicine and Toxicology Department, Faculty of Veterinary Medicine, Zagazig University, Zagazig, Egypt; ^6^Chemistry Department, Faculty of Applied Science, UmmAl-Qura University, Makkah, Saudi Arabia; ^7^Department of Pathology, Faculty of Veterinary Medicine, Alexandria University, Edfina, Egypt; ^8^Biological Application Department, Nuclear Research Center, Atomic Energy Authority, Abu-Zaabal, Egypt; ^9^Department of Biological Sciences, Zoology, King Abdulaziz University, Jeddah, Saudi Arabia

**Keywords:** SARS-CoV-2, COVID-19, phytogenic additive, phytochemicals, health, immunity

## Abstract

Coronaviruses are the causative agents of many infectious diseases in human and animals. These included severe acute respiratory syndrome (SARS), avian infectious bronchitis (IBV) in poultry, Middle East respiratory syndrome (MERS), and coronavirus disease 2019 (COVID-19) in humans. These results had considerable death burdens and negative influences on social–economic life. Since the appearance of the outbreak of the COVID-19 pandemic, continuous investigations have been carried out by researchers to find active compounds, mainly from plants, as natural sources, that could inhibit or stop the proliferation of the causative agent of COVID-19 (SARS-CoV-2). The most common symptoms caused by infections with COVID-19 can include cough, fever, and sore throat. Nevertheless, there is a shortage of active antiviral compounds for treating different strains of coronavirus. Herbal medicine is a class of medication that originates from nature and is aimed at decreasing the use of preservatives, excipients, or other additives and, consequently, lesser side effects. The rapid spread of COVID-19 infection besides the lack of knowledge about any treatments and the growing concern of the public from the virus directed us toward writing this review article in an aim to provide alternatives to the allopathic medicine use. There is a wealth of chemical diversity in the naturally existing compounds, including their antiviral activities, which may encourage their utilization as therapeutics against viral infections, including coronaviruses. The majority of publications on the herbal remedies of coronavirus, MERS, or SARS focused primarily on the use of polar compounds. These substances displayed encouraging inhibitory influences on coronavirus in humans. These include psoralidin, scutellarein, silvestrol, tryptanthrin, caffeic acid, quercetin, myricetin, saikosaponin B2, griffithsin (lectins), and isobavachalcone. Some other agents like lycorine may be useful, if the antiviral activity is obtained by concentrations below the toxic plasma levels. According to the available literatures, the most promising inhibitors of coronaviruses are polyphenolic compounds, which are small molecules with conjugated fused ring structures.

## Introduction

Since the emergence of the recent surpassing COVID-19 disease and the proclamation of the World Health Organization of it as a pandemic on March 11, 2020, the global scientific concern has been prompted. Worldwide, the pandemic has recorded, as of September 4, 2020, 26,504,030 cases, with 873,821 deaths, and the numbers continue to increase drastically^1^. Coronaviruses (CoVs) are positive-sense single-stranded ribonucleic acid (RNA), large (genome size 26–32 kb), and enveloped viruses that fit to the Coronaviridae family and *Coronavirinae* subfamily. They can infect both humans and animals (1). Coronaviruses are allocated, based on their genotypes and serotypes, into four genera. These are *alphacoronavirus* (α-CoVs), *betacoronavirus* (β-CoVs), *gammacoronavirus* (γ-CoVs), and *delta coronavirus* (Δ-CoVs) (2, 3). All identified coronaviruses that have the potential to infect humanity belong to α-CoVs and β-CoVs, including HKU1, HCoV-NL63, HCoV-229E, HCoV-OC43, MERS-CoV, SARS-CoV, and SARS-CoV-2 (2). In the last two decades, two epidemics, and one pandemic caused by β-CoVs have emerged, namely, SARS, MERS, and COVID-19, respectively (4).

A global threat emerged in Southern China in 2002 due to the emergence of SARS-CoV that infected 8,098 people with 774 mortalities recorded (3, 5). Furthermore, in 2012, a regional epidemic in the Middle East occurred as a result of the infection with MERS-CoV, causing 2,494 infections and 858 deaths (6). Recently, on December 31, 2019, a new virus that caused the emergence of COVID-19 disease has been identified in Wuhan, China, and it was named SARS-CoV-2 because nearly 70% of its genome is identical to that of SARS-CoV (7, 8). The main targets of SARS-CoV-2 medications include RNA-based RNA polymerase, papain-like protease (PL^pro^), 3-chymotrypsin-like protease (3CL^pro^), and spike glycoprotein proteins (S protein).

The spike glycoprotein protein (S protein) helps SARS-CoV-2 to invade the human cells as the entry point of the virus is the direct interaction between S proteins and human angiotensin-converting enzyme 2 (hACE2) (7, 9). As a result of the frantic worldwide race to find an effective vaccine or cure, several synthetic and natural compounds, as well as antiviral medications, have been proposed to overcome the morbidity and mortality caused by this pandemic. Chloroquine phosphate and hydroxychloroquine were primarily suggested to treat severe cases based on several of their mechanisms of action. These included alkalization of cellular phagolysosomes (10, 11). These included remdesivir, arbidol, and lopinavir as antiviral medications, peptide EK1, neuraminidase inhibitors, and nucleoside analogs which were then suggested as encouraging agents for SARS-CoV-2 (12–16).

**Figure 1:**
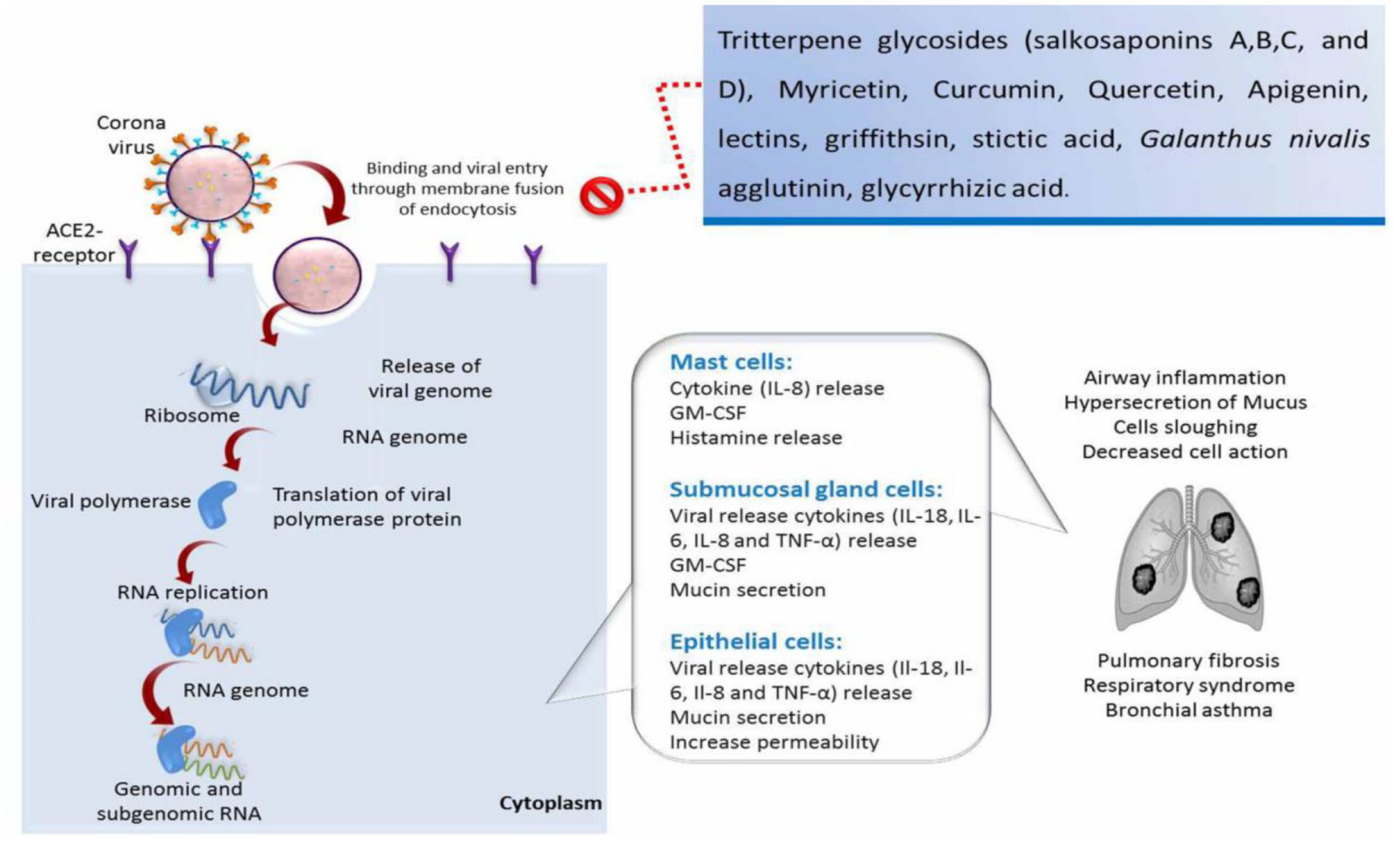
Schematic illustration of the transmission and replication cycle of coronavirus, induction of respiratory syndrome and lung fibrosis, and the natural compound inhibitory effects on viral infection. IL, interleukin; TNF, tumor necrosis factor; GM-CSF, granulocyte-macrophage colony stimulating factor.

The transmission and replication cycle of coronavirus and the natural compound inhibitory effects on viral infection is exemplified in Figure 1.

Infections of animal coronaviruses have attracted veterinary attention for nearly a century. However, in the last two decades, the role of animals in generating coronaviruses has been highlighted since the emergence of SARS, MERS, and COVID-19 diseases, which had a zoonotic origin. Hence, animal coronaviruses became important models for studying how this large family of viruses evolves, generating strains with different biological characteristics. Efforts of veterinary scientists to develop effective anti-coronavirus treatments or vaccines against well-known animal coronaviruses, using herbs and/or Western remedies, contribute to the development of potential therapeutic and prophylactic strategies against SARS-CoV-2. Plants are one of the medicinal active compound sources that have been broadly used as treatments for several diseases caused by microbes (17–19). Moreover, purified natural products and traditional herbal medicines may guide the development of more effective substances based on their structure and desired activities. There are many plant bioactive substances cited to have activities as an antifungal (20), antibacterial (21–23), and antiviral (24, 25). The naturally occurring compounds that have been recognized to have antiviral activity can be used as a starting point to find probable bioactive substance candidates against SARS-CoV-2. Molecular docking can be employed to predict how the receptor protein interacts with bioactive compounds (ligands) (26, 27). Several previous experiments have been carried out to find bioactive substances of plants that have the potential to inhibit and/or to reduce viruses' proliferation (28–30). A recent publication states that nelfinavir (a selective inhibitor of HIV protease), by using molecular docking, is a drug candidate for the possible treatment of COVID-19 by inhibiting the main protease (M^pro^) (31). This review focuses on introducing available information on phytogenic compounds or extracts that may have therapeutic potential and demonstrate antiviral capabilities to inhibit coronaviruses, hoping to help in suggesting a naturally based drug for SARS-CoV-2.

### Traditional Chinese Medicine

Traditional Chinese herbal medicine (TCHM) therapy is accomplished by mixing some Chinese herbs that have been stipulated by Chinese herbalists relying on Chinese diagnostic patterns of patients' syndrome. Several Chinese herbal prescriptions, such as *Yinhuapinggan granule, Lianhua qingwen* capsule, and San Wu Huangqin Decoction, have been suggested to control the replication and proliferation of the viral particles and may thus improve pneumonia caused by influenza viruses (32–34). WHO has invested a lot of works to find out the effectiveness of herbal medicine, since the outbreak of the SARS epidemy in 2002. Treatments of TCHM have proven to be successful in the prevention and treatment of SARS-CoV (35–37). Also, the combination of TCHM and western medicine remedies was able to reduce the complications induced by the antibiotic, glucocorticoid, and antiviral therapies (38, 39).

After the outbreak of the COVID-19 disease in China, a team of experts was commissioned by the Chinese State Administration of Traditional Chinese Medicine to evaluate the efficacy of the TCHM treatment program. The first case was a patient with TCHM with symptoms, who was cured in Beijing after treatment, which was documented on January 24, 2020. Subsequently, another patient was tested after treatment with TCHM, which prompted a wider application of TCHM for patients with SARS-CoV-2 pneumonia. On January 27, 2020, a report entitled “Diagnosis and Treatment of Pneumonia Caused by Novel Coronavirus Infection (Trial Version 4)” was issued by the Chinese State Administration of TCM and the General Office of the National Health and Health Commission of China which included the updated treatment programs of TCHM (40). These administrations required local and international health committees to implement TCHM treatments and strengthen their integration with western medicine.

### The Role of Phytochemicals in Treating Coronavirus

It is of importance to screen the potential of phytogenic compounds to participate in finding substance candidates, which are able to prevent viral infections (41). Some substances that show violations toward Lipinski's regulation of five (RO5) are epigallocatechingallate (2), pectolinarin (3), hesperidin (3), nelfinavir (1), cannabinoids (2), rhoifolin (3), and bis(3,5,5-trimethylhexyl) phthalate (1) (42). Lipinski's rule is tested to assess the drug-likeness and to determine if any specific chemical substance possesses physical and chemical characteristics to be examined as an active drug that can be orally administrated in humans (43). This rule is beneficial because it acts as a basis for the prediction of a high probability of failure or success of one substance with specific biological or pharmacological activity to be developed as a drug. This rule also suggests that if a compound shows two or more range-of-five (RO5) violations, then the substances show low solubility or permeability (44).

Dozens of proteins are coded by coronaviruses, some of which are involved in viral replication and entry into cells, including 3CL^pro^ (3-chymotrypsin-like protease; 3CL^pro^), one of the main proteases of SARS-CoV-2 (9); M^pro^, a key enzyme for replication of SARS-CoV-2 (45); and S protein, an essential binding protein for the transmission of SARS-Cov-2 through the cellular membrane via hACE2 (46). The function of 3CL^pro^ and M^pro^ together is to form a viral replication complex. Therefore, M^pro^, 3CL^pro^, and S protein are ideal targets for researchers and drug developers to design and develop an effective cure for COVID-19 (9).

Furthermore, Coutard et al. (47) suggested an inhibitor for furin, because the S protein sequence has a specific furin-like cleavage.

The inhibition activities of phytochemical agents against M^pro^ and S proteins have been cited, and some of these compounds are shown in Table 1.

**Table 1:** List of plant-derived compounds and their molecular docking analysis against M^pro^/3CL^pro^ (6LU7) and S protein (6VXX), chemical structure, oral bioavailability, and sources (42).

**Compounds**	**Chemical structure**	**PubChem CID**	**Binding energy with M^**pro**^/3CL^**pro**^**	**Binding energy with S protein**	**Oral bioavailability**	**Sources**
Epigallocatechingallate	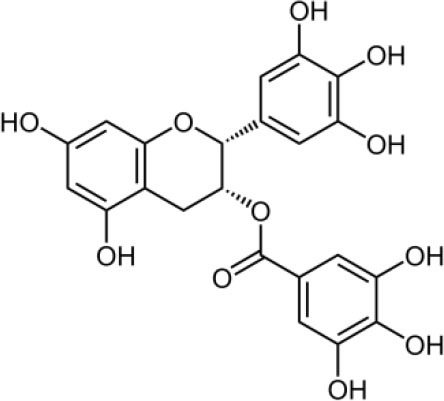	65064	−7.8	−9.8	High	Green tea (*Camellia sinensis*), hazelnut (*Corylus avellana*), onion (*Allium cepa*), plum (*Prunus domestica*), skin of apple (*Malus domestica*)
Myristicin	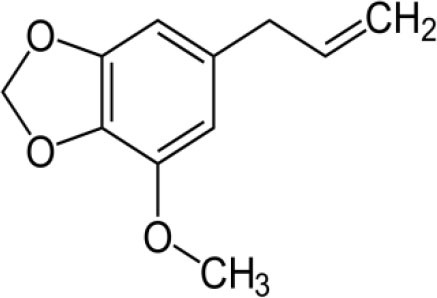	4276	−5.3	−6.1	High	Nutmeg (*Myristica fragrans*)
Cannabinoids	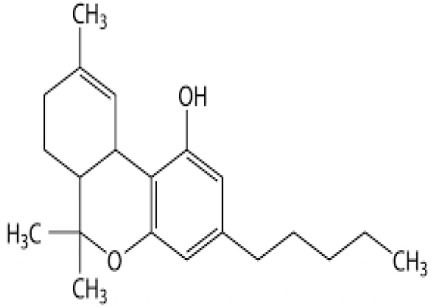	9852188	−8.0	−10.2	Low	Marijuana (*Cannabis spp*.)
Hesperidin	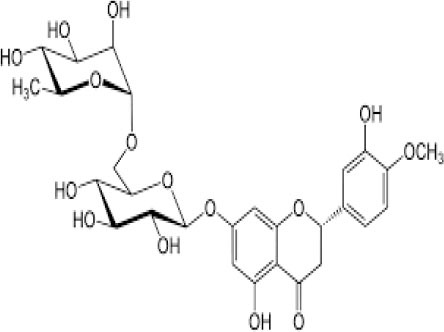	10621	−8.3	−10.4	Low	Citrus fruit (*Citrus spp*.), yellow toadflax (*Linaria vulgaris*), peppermint (*Mentha spp*.)
Rhoifolin	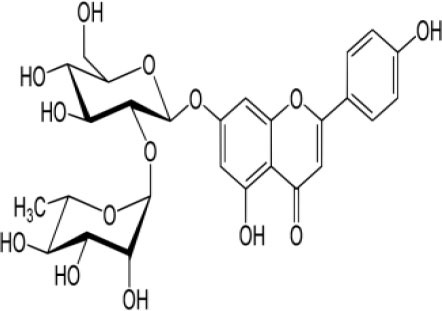	5282150	−8.2	−9.5	Low	Bitter orange (*Citrus aurantium*), grape (*Vitis vinifera*), bananas (*Musa* spp.), lemon (*Citrus limon*), grapefruit (*Citrus paradisi*), tomato (*Lycopersicon esculentum*)
Eugenol	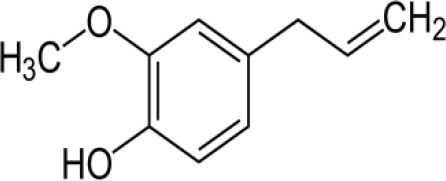	3314	−5.4	−6.1	High	Clove (*Syzygium aromaticum*)
Tangeretin	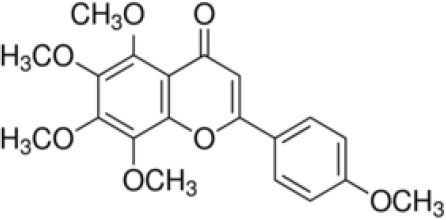	68077	−6.5	−7.9	Low	Citrus fruit (*Citrus spp*.)
Bis(3,5,5-trimethylhexyl) phthalate	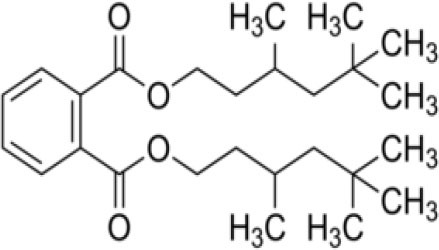	34277	−5.6	−6.1	Low	Leaf of keluak (*Pangium edule*)
Kaempferol	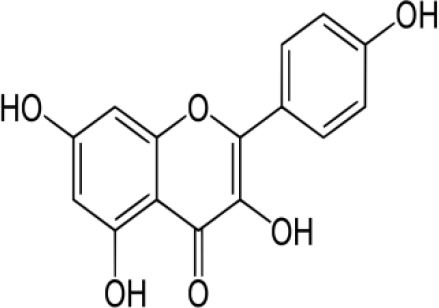	5280863	−7.8	−8.5	Low to good	Broccoli (*Brassica oleracea var. italica*), spinach (*Spinacia oleracea*), tea (*Camellia sinensis*), beans (*Phaseolus vulgaris*), Kale (*Brassica oleracea var. sabellica*)
6-Shogaol	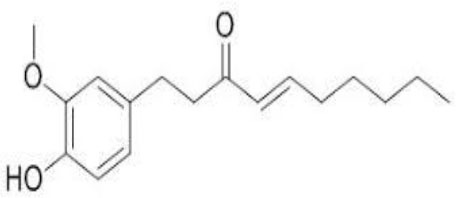	5281794	−5.8	−5.5	Low	Ginger (*Zingiber officinale*)
Chalcone	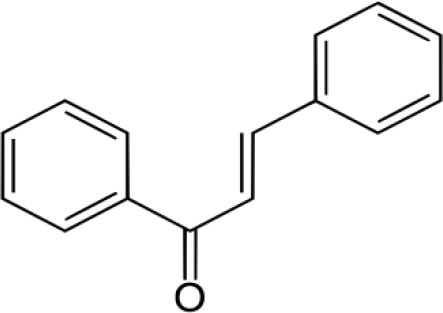	637760	−6.2	−7.5	Very good	Citrus fruit (*Citrus spp*.)
Pectolinarin	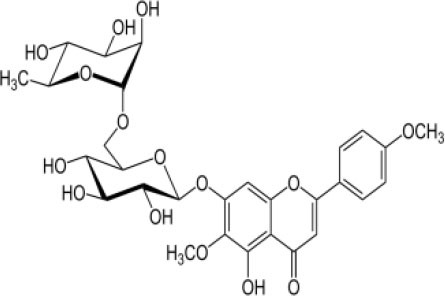	168849	−8.2	−9.8	High	Yellow toadflax (*Linaria vulgaris*), plume thistles (*Cirsium spp*.)
Morin	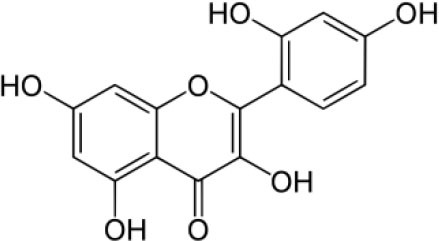	5281670	−7.8	−8.8	Very low	Guava (*Psidium guajava*), almond (*Prunus dulcis*), Osage orange (*Maclura pomifera*), old fustic (*Chlorophora tinctoria*)
Nobiletin	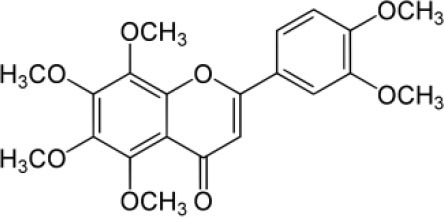	72344	−6.4	−8.1	Low	Citrus fruit (*Citrus spp*.)
Herbacetin	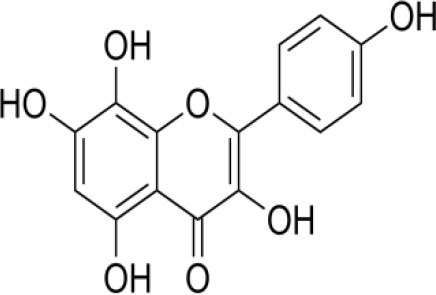	5280544	−7.2	−8.3	Good	Common boneset (*Eupatorium perfoliatum*), common horsetail (*Equisetum arvense*), Golden root (*Rhodiola spp*.), Gossypium (*Gossypium hirsutum*)
6-Gingerol	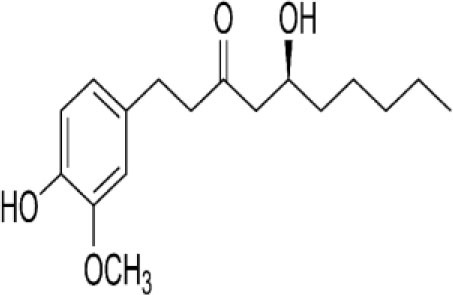	442793	−5.8	−6.3	Low	Fresh finger (*Zingiber officinale*)
Ethyl cholate	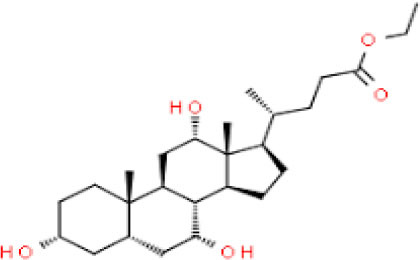	6452096	−6.7	−8.1	N/A	Leaf of keluak (*Pangium edule*)

*N/A, not applicable*.

For instance, herbacetin, epigallocatechingallate, rhoifolin, pectolinarin, hesperidin, cannabinoids, and morin showed better docking poses than did nelfinavir, hydroxychloroquine sulfate, and chloroquine (43). Nevertheless, these substances have low oral bioavailability except a few of them, i.e., pectolinarin, epigallocatechingallate, myristicin, and eugenol (Table 1). The low oral bioavailability of these substances illustrates a real problem for drug designers. The substances may have high effectiveness in *in vivo* and/or *in vitro* studies; it may fail as a new drug, when considered in clinical trials, besides the oral availability of some compounds, which may decrease if combined with food (48). Therefore, the oral bioavailability of phytogenic compounds must be taken into consideration, when predicting them as candidate drugs.

Limited studies have evaluated the potential of some plant-derived compounds for inhibiting M^pro^ and S protein of SARS-CoV-2 using the molecular docking method. These studies are essential in facilitating subsequent *in vitro* and *in vivo* investigations either in clinical trials on humans or in animal models. Hesperidin, the principal flavanone glycoside in the citrus peel, has been cited to be better than nelfinavir (49) with a docking ratio of S protein and M^pro^ ranging from −8.3 to −13.51 and −9.6 to −10.4, respectively (42, 50). Moreover, Chen et al. (51) revealed that the best hesperidin position against SARS-CoV-2 3CL^pro^ was −10.1. Also, the active compounds of *Cannabis sativa* and *C. indica*, namely, cannabinoids, recorded a docking score against S protein and M^pro^ of −8 and −10.2, respectively (42). Cannabinoids have been described as effective anti-inflammatory (52) and anti-herpes simplex virus agents (53).

Epigallocatechingallate is established in high quantity in green tea (54). The best binding position of this compound against M^pro^ was −7.8, and that against S protein was −9.8. Earlier studies have stated the ability of epigallocatechingallate to restrain the proteolytic potential of SARS-CoV 3CL^pro^ (55). Herbacetin, which can be originating from *Rhodiola* sp. (golden root), has antiviral activity against vesicular stomatitis virus, a prototype of negative-strand RNA viruses, such as rabies viruses and influenza (56). Table 2 summarizes the antiviral activities of some herbal plants and their derivatives against human coronaviruses.

**Table 2:** Antiviral activities of herbal plants and their derivatives against human coronaviruses *in vitro* (cell culture).

**Plant species**	Virus strain^*^	**Extract type**	**Bioactive compounds**	**EC_**50**_ or IC_**50**_ (μg/mL)**	**Mechanism(s) of action**	**Reference**
CTM	SARS-CoV-2	ND	Betulinic acid, coumaroyltyramine, kaempferol, lignan, desmethoxyreserpine, cryptotanshinone, dihomo-c-linolenic, dihydrotanshinone, moupinamide, N-cis-feruloyltyramine, quercetin, sugiol	ND	Reduce viral replication Inhibition of 3CL^pro^ and PL^pro^ Inhibition of cellular entry and spike protein	(57)
*Camellia sinensis*	SARS-CoV-2	ND	Theaflavin		Binding to RNA-dependent RNA polymerase	(58)
*Artemisia annua* *Lindera aggregata* *Lycoris radiata*	SARS-CoV (BJ001 and BJ006)	Ethanol extract (95%)	Lycorine in L. radiata	34.5–39.2 80.6–88.2 2.1–2.4	Not determined	(59)
*Toona sinensis*	SARS-CoV FFM1	Boiled water extract of leaves	Not determined	30–43	Not determined	(60)
*Glycyrrhizae radix*	SARS-CoV FFM1	Used chemical standards	Glycyrrhizin 18β-Glycyrrhetinic acid	365 ± 12 μM	Not determined	(61)
*Barometz* *Gentiana scabra* *Dioscorea batatas* *Cassia tora* *Taxillus chinensis*	SARS-CoV (Hong Kong strain)	Ethanol extract (75%)	Secoiridoid & glycoside Polysaccharides Emodin Quercetin	8.70 8.06 8.43 5.39	Not determined	(62)
*Urtica dioica*	SARS-CoV Urbani strain (200,300,592)	Used chemical standards	*Urtica dioica* agglutinin	2.6 ± 3.7 μg/mL	Inhibition of viral replication in a dose-dependent Binding to spike protein of SARS-CoV and N-acetylglucosamine-like residues on the glycosylated envelope	(63)
*Artemisia annua*	SARS-CoVCL^pro^	ND	Aurantiamide acetate	ND	Inhibiting the active pocket of CoV protease	(64)
*Aglaia* sp.	MERS-CoV EMC/2012	Used chemical standards	Silvestrol	1.3	Specific inhibitor of RNA helicase eIF4A	(65)
*Broussonetia papyrifera*	MERS-COV PL^pro^	Ethanol extract	Kazinol F Broussochalcone A	39.5 ± 5.1 42.1 ± 5.0	Non-competitive inhibition of CoVPL^pro^	(66)
*Stephania tetrandra*	HCoV-OC43	Used chemical standards	Tetrandrine Fangchinoline Cepharanthine	0.33 ± 0.03 ± 0.07 0.83 ± 0.07	Inhibit viral S and N proteins expression and viral replication	(67)
*Strobilanthes cusia* leaf	HCoV-NL63	Methanol extract	Tryptanthrin Indigodole B	0.06 2.09	Blocking activity of papain-like protease 2 and viral RNA genome synthesis	(68)

* *SARS-CoV-2 results obtained by computer modeling. CTM, Chinese traditional medicines; CLpro, chymotrypsin-like protease; PLpro, papain-like protease; ND, no data*.

It has also been cited that herbacetin might act as an inhibitor of MERS-CoV/3CL^pro^ (69). The docking scores of this compound against S protein, M^pro^, and SARS-3CL^pro^ were −8.3, −7.2, and −9.263, respectively (42, 70). The same authors recorded the docking score (a mathematical equation employed to approximately establish the binding affinity between two molecules after they have been docked) of rhoifolin, a flavon found in fresh leaves of *Rhus succedanea* and Citrus grandis, against S protein, M^pro^, and SARS-CoV 3CL^pro^, and it was −9.5, −8.2, and −9.565, respectively. The induced-fit docking results of pectolinarin and morin against SARS-CoV 3CL^pro^ were −8.054 and −8,930 (70), respectively; however, the best pose between these compounds and S protein was −9.8 and −8.8, and −7.8 and −8.2 with M^pro^, respectively (42). Pectolinarin can be found in plume thistles (*Cirsium* spp.), while almond, guava, and old fustic contain a high quantity of morin. The best position of kaempferol, a phytochemical found in spinach and kale, against S protein and M^pro^ was −8.5 and −7.8 (41)), while −8.526 was the best binding position of this compound against SARS-CoV 3CL^pro^ (70).

### Virtual Screening of SARS-CoV-2 Inhibitors

Due to the limited time, since the emergence and spread of the COVID-19 pandemic, studies conducted on SARS-CoV-2 are minimal; however, several computer modeling studies for screening purposes are available (57, 58). Despite the difficulty of obtaining consistent output through various modeling approaches, computer modeling nonetheless provides the opportunity to compare the relative binding affinity of a molecular bank concerning the intended receptor (71–73). Computer modeling may also be of specific value due to its speed and versatility. This precipitates the prospect of finding a potent inhibitor of SARS-CoV-2 instead of relying on the conventional physical selection of large banks for bioactivity of plant extracts or phytochemicals. This consumes a long time and high costs (74). The highlighted compounds by computer modeling can then be redirected to cell-based tests to evaluate their *in vitro* toxicity and efficacy, before being used in animal model experiments or human clinical trials. For instance, among 83 compounds found in TCHM virtually screened by Lung et al. (58), flavin, an antioxidant polyphenolic compound, has been identified as a potential inhibitor against SARS-CoV-2 RNA-dependent RNA polymerase.

**Figure 2:**
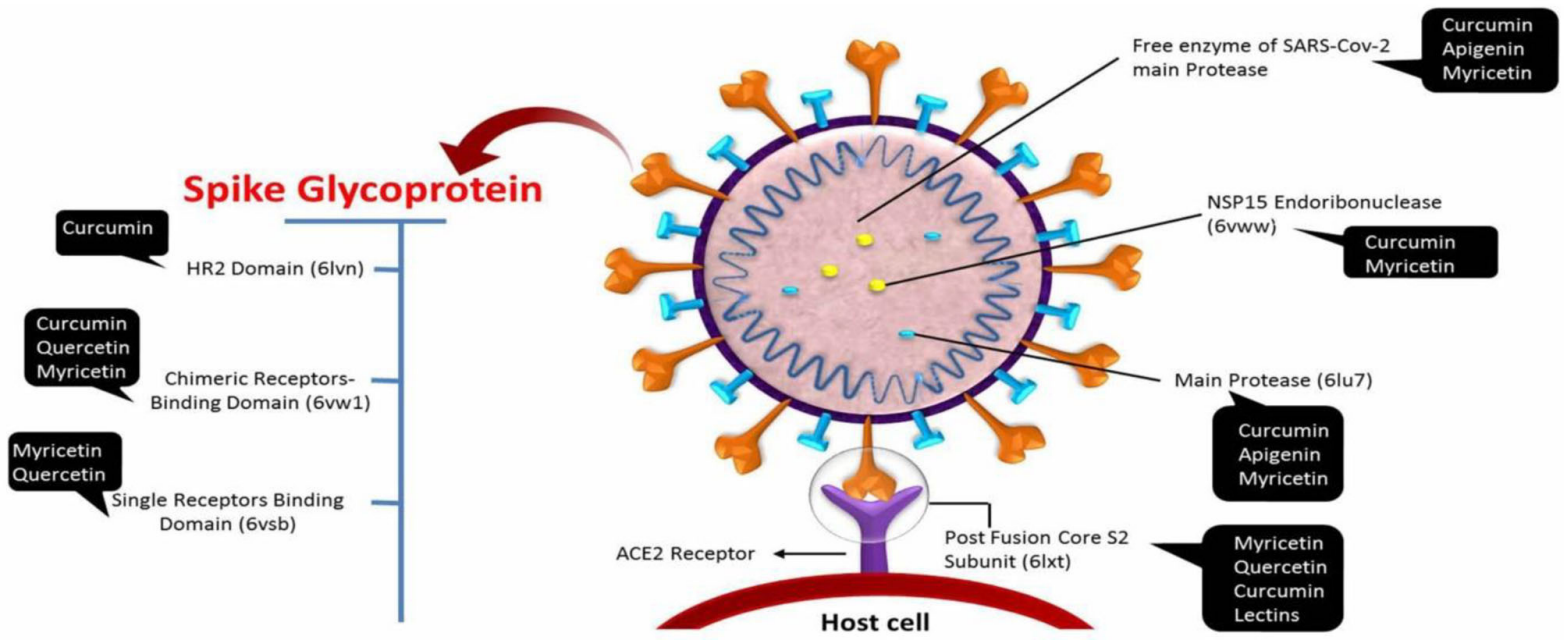
The major drug able targets of SARS-CoV-2.

Moreover, Zhang et al. (57) selected 13 compounds for further investigations as potential inhibitors for SARS-CoV-2 of 115 compounds tested in TCHM that have been virtually tested. Many of these polyphenol candidates, such as quercetin and kaempferol, are naturally present and can be considered as treatments for other diseases (75, 76). Nevertheless, there are many doubts about the potential lack of efficacy of some polyphenols and traditional herbal medicines that are promoted to treat the symptoms of COVID-19. For example, however, the TCHM called Lianhuaqingwen demonstrated antiviral potential against SARS-CoV-2, with quite higher EC_50_ when compared to the commercial drug remdesivir (~411 vs. 0.39 μg/mL, respectively) using the same method (77). The major polyphenolic drugs able targets of SARS-CoV-2 are depicted in Figure 2.

### SARS-CoV Inhibitors

**Figure 3:**
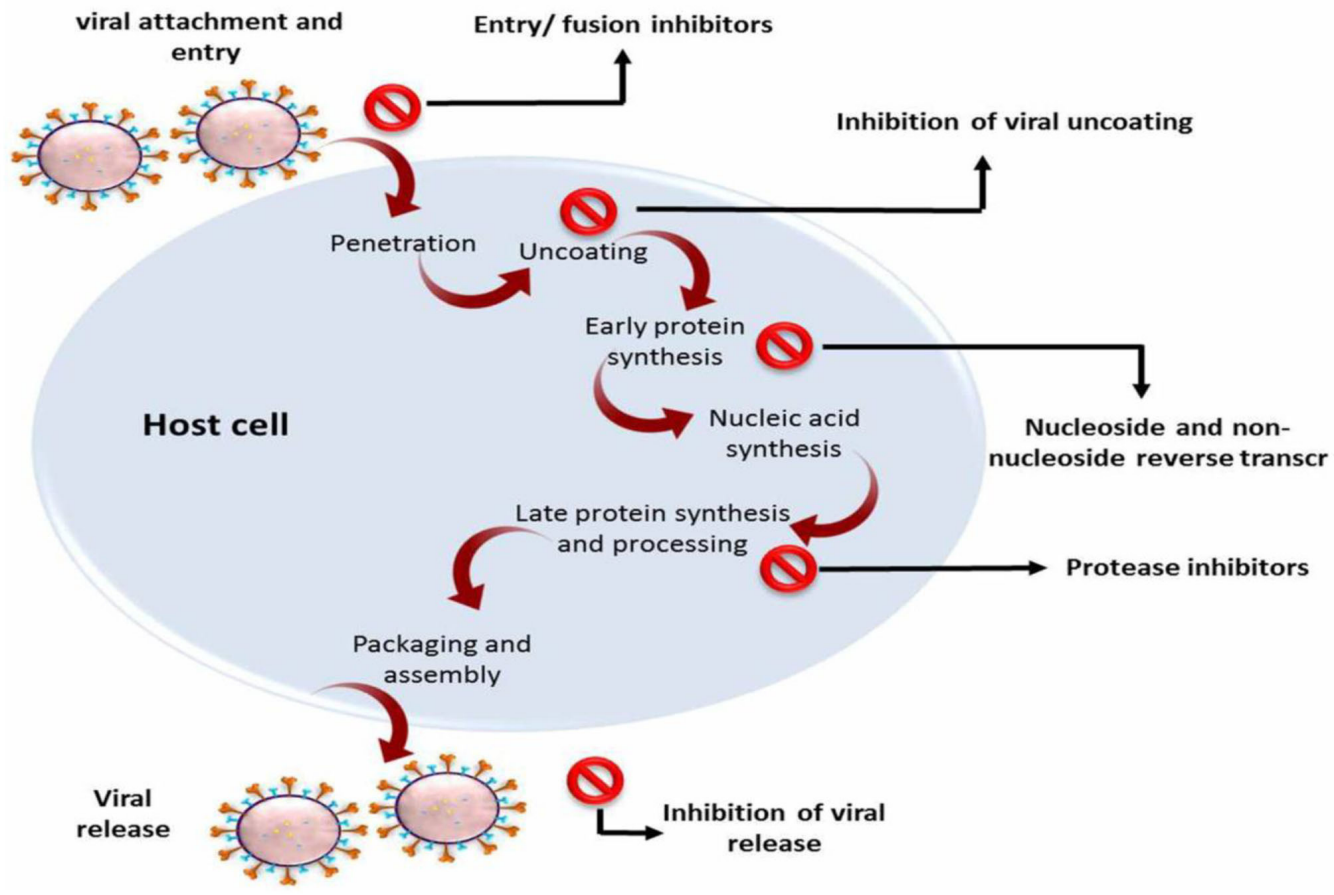
The major sites of antiviral drug action.

Due to the limited available publications on COVID-19, some publications and research articles that have been searching for SARS-CoV inhibitors can represent a good starting point to identify the competency antiviral agents against SARS-CoV-2. Therefore, it is necessary to review the antiviral agents that were successful in inhibiting this viral strain and the possibility of their effectiveness against this novel coronavirus. The primary sites of antiviral drug action are clarified in Figure 3.

#### Virtual Screening

Virtual computer docking models have been used by several investigators to screen the inhibition activities for prospective phytochemical agents against several binding sites of coronaviruses, including S protein, RNA-dependent RNA polymerase, papain-like protease (PL^pro^), and 3CL^pro^ (64, 78, 79). The authors highlighted the antiviral potential of several substances such as sabadinine and aurantiamide acetate as these compounds could inhibit or bind to critical proteins found in SARS-CoV.

#### Compound Library Screening

It is noteworthy that searching for natural phytochemicals that have inhibitory activity against SARS-CoV using extensive *in vitro* screening studies is similar to the search for a “needle in a haystack” (59, 80), despite the existence of many such studies. For instance, in the study of Li et al. (59), the authors found that there are only four of Chinese medicinal herbs (*Artemisia annua, Pyrrosia lingua, Lycoris radiata*, and *Lindera aggregata*) with moderate to high antiviral efficiency, when they examined more than 200 chloroform or ethanol extracts of these plants. Despite this low number, only one compound (lycorine) with high antiviral efficacy (EC_50_ of 15.7 ± 1.2 nM) derived from *Lycoris radiata* was allotted as a SARS-CoV prospective medication candidate (59). It is disappointing that lycorine can exert toxicity impacts at relatively low dosage levels (~1,000 μg/kg in dogs), and this is what the authors did not mention (73, 81).

SARS-CoV helicase (nsP13) is an enzyme that exerts a vital role in the replication, transcription, and translation of the viral genome. Minimal investigations have studied the efficacy of naturally occurring compounds to inhibit this enzyme. Yu et al. (82) screened the activity of 64 natural substances against nsP13. They indicated that the most promising candidates were the polyphenolic substances scutellarein (IC_50_ 0.86 ± 0.48 μM) and myricetin (IC_50_ 2.71 ± 0.19 μM), without any considerable noticeable toxic impact on the normal (non-tumorigenic) epithelial cells of the breast. Both compounds were noticed to inhibit nsP13 by inhibiting the activity of ATPase indirectly. Scutellarein has been conventionally considered as an anti-inflammatory agent and used in respiratory infection medication and can be isolated from Chinese Skullcap (*Scutellaria baicalensis*) (83). Myricetin is cited in vegetables such as garlic and *Calamus scipionum* and fruits such as cranberry in rationally high concentrations (84, 85).

#### Phenolic Compounds

Polyphenols are a class of mainly natural compounds that have shown antiviral properties across several investigations. For instance, the effectiveness of *Psoralea corylifolia* seeds against SARS-CoVPL^pro^ has been ascribed to the bioactive agents of ethanolic extract of this plant (polyphenolic compounds), in a bioassay-guided fractionation way (48). From the ethanolic extract, six polyphenols that vary widely in their antiviral activities (IC_50_ values between 4,200 and 38,400 nM) were isolated and identified as neobavaisoflavone, corylifol A, bavachinin, 4′-O-methylbavachalcone, isobavachalcone, and psoralidin. Isobavachalcone and psoralidin demonstrated the highest antiviral activity. When mixed, both compounds showed reversible inhibition of PL^pro^ through their ability to attach to the free form of the enzyme preferentially, rather than the complex substrate of the enzyme (type I mechanism) (48).

#### Quercetin

Quercetin is a flavonoid that originated in many fruits, grains, and vegetables, but high levels of the active substance were found, particularly in certain herbs and berries (86, 87). Several animal and laboratory investigations showed that quercetin may have the potential to inhibit a broad spectrum of viruses, including SARS-CoV, while quercetin showed a reasonable activity in inhibiting SARS-CoVPL^pro^ (IC_50_ of 8.6 ± 3.2 μM) (66) and SARS-CoV helicase (82). This could be ascribed to its chemical arrangement that is structurally similar to polyphenolics scutellarein and myricetin (82). Furthermore, the potential of quercetin to inhibit viruses may be due to its ability to restore levels of several antioxidants in body tissues. For example, quercetin has shown restoring influence by decreasing the concentrations of several antioxidants in the lungs, including catalase, reduced glutathione, and superoxide dismutase, in mice treated with influenza, suggesting that quercetin taken along with viral infection may safeguard lung tissues and preserve antioxidant capacity (88). Although providing quercetin orally simultaneously with viral installation did not restore the decrease in the level of vitamin E connected with a viral infection, it augmented the pulmonary levels of superoxide dismutase, reduced glutathione, and catalase (88).

#### Lectins

Plant lectins are naturally occurring proteins that are able to combine reversibly and correctly to carbohydrate groups and show antiviral activity against several viral infections, including herpes simplex virus, Ebola, influenza, and SARS-CoV (89–92). Curiously, Michelow et al. (90) reported that increasing recombinant human mannose-binding lectin levels in the plasma of mice permitted them to overcome different viral infections except the fatal Ebola. Petersen et al. (93) demonstrated that lectins had shown reasonable tolerability in clinical experiments. Moreover, a total of 33 plant lectins have been tested using a cytopathic effect assay for their antiviral activity against SARS-CoV (94). The authors noticed that EC_50_ values for *Lycoris radiata* agglutinin were low (0.45 ± 0.08 μg/mL) and did not influence the virus replication. The exact mechanisms of action for the potential antiviral activity of plant lectins were not established. Therefore, further investigations are required to prospect for a treatment of coronavirus infection among the promising classes of naturally derived substances.

#### Chemical Modification of Phytochemicals to Increase Their Potency

As described earlier, several natural derivatives demonstrate considerable promising results as SARS-CoV inhibitors; however, only few fulfill the requirements of the commercial drug in terms of selectivity and efficacy. Hence, increasing the effectiveness and antiviral activity of these compounds to the appropriate levels for therapeutic applications may require adjusting their chemical structure. Moreover, drug developers can reduce timelines and increase their chances to find effective viral inhibitors starting from understanding the structural conformity of the naturally derived compounds. Working on this principle, modification of the main active constituent of liquorice, namely, glycyrrhizin, which has been used in patients treating SARS-CoV (95), might enhance its viral inhibition activity (61). For instance, tomentins A–E (all compounds occur naturally) exhibited substantial inhibitory potential against SARS-CoV *in vitro* comparison to their non-geranylated precursor substances (96).

Moreover, quercetin has demonstrated antiviral activity against porcine epidemic diarrhea virus (PEDV) less 100 times than quercetin-7-rhamnoside (97). The antiviral activity of glycyrrhizin was also raised by 10-fold by adding 2-acetamido-β-*D*-glucopyranosylamine to its glycoside chain. The enhancement in the antiviral activity can be ascribed to increasing its attraction to the S proteins. Again, *in vivo* or *in vitro* route of administrations and duration of the applications should be given. Furthermore, glycyrrhizin conjugation with free COOH and amino acid residues increased its activity up to 70-fold, although with a significant decrease in selectivity (73). Although the examples of amendments displayed here differ, it is necessary to not assume that substitutions were randomly supplemented but rather performed to target a particular biochemical pathway and/or receptor. For example, the supplementation of residues of amino acids and glycosides to glycyrrhizin was conducted in request to raise its attraction to the highly glycosylated S proteins of SARS-CoV. Consequently, future investigations seeking to find effective natural inhibitors of SARS-CoV-2 must also take into account investigating the potential designing or modifications of synthetic derivatives that might enhance required activity.

### MERS-CoV Inhibitors

Few investigations have been carried out to determine the therapeutic potential of plant-derived compounds against MERS-CoV. Müller et al. (65) *in vitro* noticed that the replication of MERS-CoV was significantly inhibited by a phytochemical derived from *Aglaia* sp. called silvestrol. The inhibition of viral replication caused by silvestrol can be recognized to its inhibitory activity of RNA helicase eIF4A, which prevents protein expression of coronavirus and preclude the establishment of replication and transcription complexes (65). Also, the protein obtained from the red algae *Griffithsia*, which is called griffithsin, can be considered an appropriate inhibitor of MERS-CoV. Griffithsin can inhibit the attachment of MERS-CoV and several human coronaviruses to host cells by specifically binding to glycans of protein spikes of the viruses via its three carbohydrate-binding domains (98, 99). It is worth noting that griffithsin has a specificity index against human coronavirus cells (compared to human fibroblast or colorectal adenocarcinoma cell lines) established between 30 and 3,100 (98). Therefore, griffithsin can be considered an appropriate candidate for clinical studies as well as animal model experiments against SARS-CoV-2.

### Inhibitors of Other Human Coronaviruses

Several plant-derived compounds have been tested *in vivo* for their antiviral potential against human coronavirus strains. Saikosaponins (A, B2, C, and D) characterize a group of oleanane derivatives, usually existing as glycosides, which naturally occur in medicinal plants such as *Scrophularia scorodonia, Heteromorpha* spp., and *Bupleurum* spp. The antiviral potential of saikosaponins has previously been evaluated against several viruses such as measles virus and hepatitis B virus (100, 101), hepatitis C (102), and HCoV-229E coronavirus strain (103). Saikosaponins (A, B2, C, and D) showed good to moderate antiviral potential against HCoV-229E, particularly saikosaponin B2, which showed the highest efficacy (EC_50_ of 1.7 ± 0.1 μM). The same compound showed inhibitory activity on viral attachment and penetration. It seems that saikosaponin B2 has the potential to display a wide range of bioactivity, including inhibition of cellular entry of hepatitis C as well as inhibition of drug transporters that are associated with resistance of multidrug and present on the cellular surface (102, 104).

The primary bioactive naturally occurring compounds identified in *Stephania tetrandra* and associated species have been scanned for their antiviral activities against HCoV-OC43 (67). These compounds were cepharanthine, tetrandrine, and fangchinoline, and all of them exhibited antiviral activity with EC_50_ values of 0.83 ± 0.07, 0.33 ± 0.03, and 1.01 ± 0.07, respectively (67). The ability of these compounds to inhibit viral-induced apoptosis might be attributed to their potential to suppress the virus-induced host response, virus replication, and viral S protein and nucleocapsid protein (N protein) expression. Moreover, tetrandrine can improve the host response induced by the virus by activating the p38 MAPK pathway in MRC-5 cells.

The viral product of another human coronavirus strain HCoV-NL63 has been decreased (EC_50_ of 1.17 ± 0.75 μg/mL) by treatment of the ethanolic extracts of *Sambucus formosana* stems (105). Caffeic acid was the most potent antiviral agent (EC_50_ of 3.54 ± 0.77 μM; or ~640 ± 140 ng/mL) identified among the extracts' phenolic compounds. It has been cited that caffeic acid inhibits the attachment of HCoV and other viruses such as hepatitis B to host cells by the ability to bind to S proteins. Still, the specific binding sites were not identified (106). Notably, extracts from *S. formosana* are likewise non-toxic and suitable for human use as the extracts of *Sambucus nigra*, another species that belongs to the same genus of *S. formosana*, have been commercialized to treat symptoms of colds and flu. However, clinical studies would be necessary to ensure this. The bioavailability and delivery mechanisms of these extracts must also be taken into consideration to attain therapeutic plasma levels for viral inhibition (107).

The antiviral potential of *Strobilanthes cusia* leaf's methanolic extract against HCoV-NL63 has been recently stressed by Tsai et al. (107), and the authors have noticed an efficient reduction in virus yield in infected cells (EC_50_ = 0.64 μg/mL) in a dose-based manner. The six substantial phytochemicals of the extract have been isolated, purified, and identified, and among them, indigodole B, an indole alkaloid derivative, and tryptanthrin, a natural alkaloid having the basic indoloquinazoline moiety, exhibited the highest antiviral activity (EC_50_ of 2.60 and 1.52 μM, respectively) against HCoV. The rise in the antiviral potential of tryptanthrin compared to indigodole B may be due to the occurrence of the double bond at C5a in tryptanthrin, as compared to the supplemental ethyl moiety in indigodole B. Thus, the supplementation of a double bond in the quinazoline ring for substances based on structural conformity for tryptanthrin could substantially elevate their antiviral activity. Furthermore, tryptanthrin has also shown a wide range of biological activities, including anti-allergic, antioxidant, anti-inflammatory, anti-protozoal, antimicrobial, and anticancer action (108). Tryptanthrin has also been observed to prevent the early and late replication periods of HCoV-NL63 by inhibiting the post-entry replication stage of HCoV, blocking papain-like protease two activity, and the viral RNA genome synthesis (68). As the spike protein of HCoV-NL63 targets hACE2 receptors such as SARS-CoV and SARS-CoV-2, this indicates a high structural similarity and conserved sequence between these viruses (109). Thus, tryptanthrin has a high latency to be assessed as a potential anti-SARS-CoV-2 agent.

Finally, besides the antiviral potential of griffithsin and silvestrol against MERS-CoV, as mentioned earlier, they also appear to have inhibition impacts against other human coronaviruses. Griffithsin has demonstrated high antiviral impacts (EC_50_ of 0.0032–0.33 μM) against several HCoV strains (99), and silvestrol inhibited (EC_50_ of 3 nM) HCoV-229E protein translation (65). A follow-up study attributed the inhibition potential of silvestrol to HCoV-229E in an *ex vivo* bronchial epithelial cell system to the inhibition of RNA helicase eIF4A (110).

### Animal Coronaviruses

**Figure 4:**
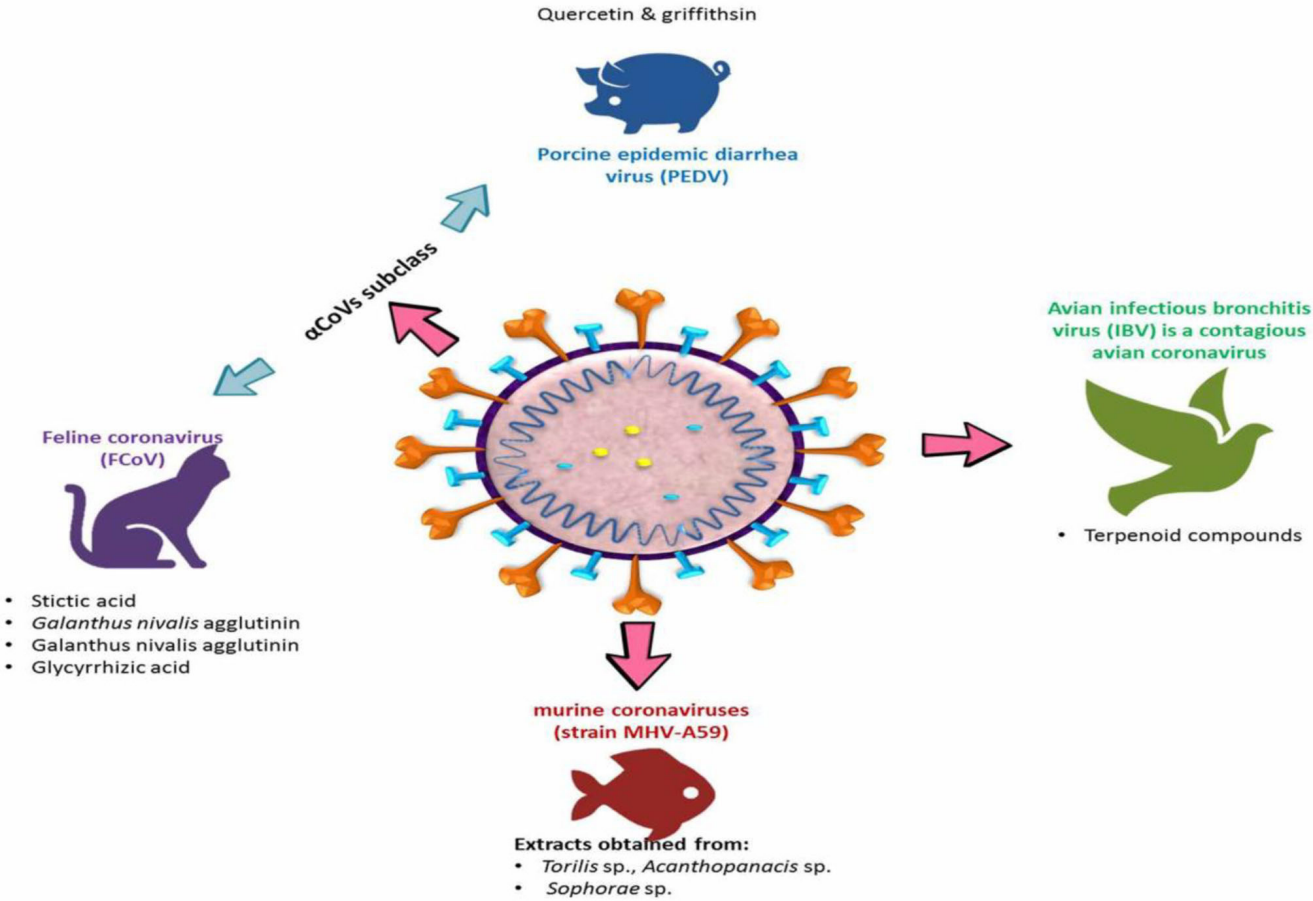
Different strains of coronavirus in animals.

A broad spectrum of livestock and domestic animals suffers from severe morbidity and mortality caused by many strains of animal coronavirus, leading to a sizeable economic demise around the world (111, 112). Coronaviruses are considered by their ability to adapt and rapidly mutate in addition to their genomic diversity, and these characteristics represent a unique challenge for the development of new antivirals. Therefore, it is important to explore alternative ways to control these viruses that can be effective across many or all serotypes. Several of the natural plant-derived substances have shown high antiviral activity against several animal coronaviruses (Table 3) through one or more of these mechanisms: (1) stimulation of the immune response of the host (123), (2) the direct inhibition of some viral parts such as spike proteins or proteases as abovementioned in the method of antiviral impacts of silvestrol and griffithsin (65, 98), and (3) host-targeting forming antiviral substances, thus blocking the virus entry to the host's cell, for example, inhibition of the clathrin-based endocytosis pathway by extracts of *Cinnamomi* sp. that avoid the virus entry to the host cells for instance (124). Different strains of corona virus in animals are illustrated in Figure 4.

**Table 3:** Antiviral activities of herbal plants and their derivatives against animal coronaviruses.

**Plant species**	**Virus strain**	**Extract type**	**Bioactive compounds**	**EC_**50**_ or IC_**50**_ (μg/mL)**	**Mechanism(s) of action**	**References**
*Ginkgo biloba*	PEDV CV 777	Ethanol extract (98%)	Polysaccharide mixture	1.7 ± 1.3	Inhibits viral attachment and entry steps in a dose-dependent manner	(113)
*Griffithsia* sp.	PEDV (NJ-PEDV)	Used purified compound	Griffithsin	~0.08 μM	Prevents viral attachment to host cells	(114)
*Houttuynia cordata*	PEDV CV 777	Methanol extract	Quercetin Quercetin 7-rhamnoside Luteolin Apigenin Possibly polyphenols	~ 5.6 ± 2.6 μM ~ 0.03 ± 0.01 μM ~ 0.7 ± 0.7 μM ~ 0.4 ± 0.4 μM 1.95	Not determined	(97)
*Amelanchier alnifolia* *Rosa nutkana*	BCV	Methanol extract	*A. alnifolia*: possibly prunasin *R. nutkana*: ND	<200 <200	Not determined	(115)
*Alstonia scholaris*	Avian IBV	Ethanol extract (50%)	Alstotide 1 Alstotide 3	35 μM 55 μM	Interferes with spike proteins and membrane	(116)
Eucalypts and several other plants	Avian IBV Gray strain	Used chemical standard	Eucalyptol (1,8-cineole)	0.61 ± 0.7 mM	Incompatible with the association between IBV nucleocapsid protein and RNA	(117)
*Sambucus nigra*	Avian IBV Beaudette strain	Ethanol extract (70%)	Possibly lectins or flavonols	ND	Compromises membrane integrity and disrupts virion structure	(118)
*Galanthus nivalis*	FCoV NTU156	Used commercial standard	*Galanthus nivalis* agglutinin	0.0088 nM	Binds to membrane proteins and spike proteins	(119)
Lichen and several other plant species	FCoV (FIPV1146)	Used commercial standard	Quercetin 7-rhamnoside 7-Methyl luteolin Steviol 7-benzyl luteolin	77.2 ± 13.8 28.5 ± 4.2 500 > 500	Inhibition of 3CLpro	(120)
*Sophorae* sp. *Sanguisorbae* sp. *Torilis* sp. *Acanthopanacis* sp.	MHV-A59	Methanol extract	Not determined	0.8 ± 0.2 3.7 ± 1.4 0.8 ± 0.0 (μg/mL) 0.9 ± 0.1	Inhibition of protease activity or RNA-dependent RNA polymerase	(121)
*Punica granatum*	MHV-A59	Ethanol/water extract	Possibly polyphenols	≥ 200 μg/mL	Interact with surface glycoprotein spikes	(114)
*Nigella sativa* *Anthemis hyaline* *Citrus sinensis* *Ziziphus jujuba*	MHV-A59	Ethanol extract	Not determined	ND	Inhibition of viral replication	(122)

*PEDV, porcine epidemic diarrhea virus; IBV, (avian) infectious bronchitis virus; BCV, bovine coronavirus; FCoV, feline coronavirus; MHV, mouse hepatitis virus; ND, no data*.

### Porcine Epidemic Diarrhea Virus

Porcine epidemic diarrhea virus (PEDV) is a severe enteric coronavirus from the α-CoV subclass that infects the cells lining pigs' gastrointestinal tract causing severe dehydration and diarrhea, leading to economic losses in porcine herds worldwide. Many natural phytochemicals have demonstrated antiviral activities against PEDV, particularly quercetin and griffithsin and their derivatives that have also been considered as antiviral agents against human CoVs (42, 65, 98). It is worth noting that Zhang et al. (57) reported some chemical derivatives that can inhibit SARS-CoV-2 proteases, and quercetin was shortlisted. Furthermore, quercetin-7-rhamnoside at a low concentration of 0.03 μM provided 50% inhibition of viral activity, lower than that of quercetin about 187 times (97), and its specificity was remarkably high (*SI* = 7,143), which is proposed for future clinical trials as well as animal model experiments against SARS-CoV-2. The authentic mode of actions of quercetin-7-rhamnoside or quercetin against PEDV was not yet determined; however, a previous computer modeling study conducted by Zhang et al. (57) has indicated that silico testing (a computer model to complement and accelerate *in vivo* and *in vitro* practices) of these compounds inhibits and binds to the SARS-CoV proteases 3CL^pro^ and PL^pro^. These studies emphasized the significance of considering all potential isomers of a compound to identify the most biologically active chemical species.

In general, several studies have emphasized that naturally existing substances active against animal coronavirus strains (e.g., quercetin, griffithsin) are also active against human coronaviruses. This confirms the possibility of using natural-derived substances with recognized antiviral activity to identify drugs for the treatment of animal coronavirus strains of economic importance, such as PEDV. However, it is of concern to test multiple strains of coronavirus when searching for the antiviral potential of phytochemicals and to determine their precise mechanism of effect to develop effective drugs against SARS-CoV-2.

### Coronavirus in Cats

Feline coronavirus (FCoV) is another coronavirus that belongs to the α-CoV group that causes a severe cat disease and without actual antiviral medication available yet. A computer screening study was conducted by Theerawatanasirikul et al. (120) for a library of naturally occurring compounds against a mutated form of FCoV called feline infectious peritonitis virus (FIPV). The authors, firstly, virtually *in vitro* screened the potential binding of tested phytochemicals to FIPV 3CL^pro^. Then, they used a protease inhibitor assay against the same enzyme to evaluate the 15 most promising compounds in *in vitro* trials. Among the tested compounds, no inhibition (IC_50_ > 500 μM) was observed by steviol and 7-benzyl luteolin; however, moderate inhibition (IC_50_ of 77.2 ± 13.8 μM) was demonstrated by quercetin 7-rhamnoside whereas 7-methyl luteolin and stictic acid showed the lowest IC_50_ values (28.5 ± 4.2 and 29.4 ± 4.6 μM, respectively). While, when the authors used the cytopathic effect assay to test these active compounds, only stictic acid showed a protective impact on viral-induced apoptosis. Furthermore, Hsieh et al. (119) considered *Galanthus nivalis* agglutinin as a potent inhibitor of FCoV, with high selectivity index (>218) and EC_50_ of just 0.0088 nM, through binding to its membrane and spike proteins, and prevented its attachment to the host cells. The authors reported that this mannose-binding lectin outperformed all comparable synthetic antiviral agents.

Another screening study against 19 substances was conducted by McDonagh et al. (125), and the authors focused on naturally occurring compounds that have been observed to have antiviral impacts against coronavirus strains or other RNA virus strains. They used many phytochemicals, including hesperitin, artemisinin, baicalin, glycyrrhizic acid quercetin, hesperidin, curcumin, and rutin at single doses ranging from 10 to 50 μM. Although none of these agents reached EC_50_, glycyrrhizic acid at a level of 25 μM was the most promising, causing a decrease in cytopathic influence assay by 26.7%. Despite these findings, further researchers are required to examine more concentrations of these compounds against FCoV.

### Murine Coronavirus

Several medicinal herbal extracts were examined *in vitro* against coronaviruses in mice (strain MHV-A59) but without determining the particular substances responsible for virus inhibition (121, 126). However, based on the abundance in the tested extracts, the authors proposed potential compounds or classes of compounds that may be responsible for this impact. The significant inhibitory potential was observed to the extracts acquired from *Torilis* sp., *Acanthopanacis* sp., and *Sophorae* sp. when tested at low concentrations (<1 μg/mL). Specifically, the extract of the *Sophorae* sp. root exhibited high viral specificity (*SI* = 696), suggesting that it could be powerfully selected for future investigations aiming to screen and identify compounds that have antiviral potential. The authors suggested that the inhibition of the activity of proteases, such as RNA-dependent RNA polymerase, might be the potential mechanism of these three species to exert their antiviral activities (Table 3).

### Avian Coronavirus

Avian infectious bronchitis virus (IBV) is a contagious avian coronavirus that influences the respiratory tract, reproductive systems, gastrointestinal tract, and kidney of chickens, causing avian infectious bronchitis disease. Several studies have tested the antiviral potential of plant-derived extracts against IBV (112, 116, 127). The main mechanisms of action of viral inhibition that have been recognized in all studies on IBV appear to be through interference with the spike protein or viral envelope disruption. However, terpenoid compounds (-)-β-pinene, (-)-α-pinene, and 1,8-cineole have an exceptional mechanism to inhibit IBV and breaking its replication cycle via binding to N protein of the virus, thereby preventing its interference with the viral genomic RNA (117, 128). The structural models of the terpenoids supported the previous observations as it showed that these substances can actively connect to five amino acid residues at the active site at the N terminus of the N protein (TyrA140, TyrA92, As A138, PheA137, and ProA134) (117, 128). Yang et al. (117) reported that these amino acid residues are considerably preserved among avian coronavirus strains. Therefore, terpenoids/isoprenoids, organic chemicals derived from terpenes, can be considered as potentially strong antiviral agents against almost all IBV strains and as a reasonable target for further investigations.

The coronavirus envelope protein (E protein) is another main key target for many investigators. The E protein of avian IBV is integral to the life cycle of the virus at different stages, so any disruption or inhibition of it may lead to the elimination of these viruses.

Phytochemicals and medicinal herbal extracts may be useful in this role. Chen et al. (118) noticed that treatment with *S. nigra* extract inactivated two eminent enveloped viruses (influenza and avian IBV). The authors suggested that the extract of *S. nigra* may have the possibility to exhibit antiviral impacts against a broad spectrum of other enveloped viruses, which can be attributed to the potential synergistic effect among the extract's inhibitory compounds, particularly flavonoids and lectins. Enhancing innate immunity against SARS-CoV-2 is essential in the absence of efficacious drugs and/or vaccine treatments. In this regard, animal experiments may be a useful model to test the effectiveness of medicinal, herbs, and spice products as antiviral agents.

### Past Experiences, Current Situations, and Future Strategy

The current review presents an overview of the present knowledge and existing advances related to herbal plants and natural phytochemicals as potential agents against SARS-CoV-2 and COVID-19. Although several drugs, vitamins, minerals, and immune-stimulators are in full use to reduce the mortalities and provide appropriate medical care for the infected persons, the search for active and specific therapeutic agents is required as soon as possible. Furthermore, several agencies, companies, and research institutes, as well as different health organizations and WHO, are on the way to find and develop a vaccine against COVID-19. Currently, with the appearance of the COVID-19 pandemic, continuous investigations have been tried by scientists to find active compounds from natural sources such as plants that could inhibit and/or stop the virus replication. However, the strategy of control, prevention, and eradication of the infection of COVID-19 needs collaboration to effectively apply social distancing and precautions to reduce or stop the further spread of COVID-19. Control of the COVID-19 outbreak and future epidemics requires global efforts among clinicians, immunologists, nutritionists, researchers, veterinarians, pharmacists. Also, public awareness should be focused on the role of nutrition, medicines, spices, herbs, and natural phytochemicals, enhancing the immune system and thus health. In addition, recent published trials by Ulasli et al. (122) and Oh et al. (129) indicated that a promising role of herbs and spices in enhancing immunity incorporation of spices into daily diet may help reduce postprandial inflammation and concurrently attenuate chronic low-grade inflammation *in vitro*. In addition, in a clinical trial, El Sayed et al. (130) found that honey, Nigella sativa, and clove could cure people with COVID-19. Nonetheless, research is needed to identify the candidates' agents and examine their effectiveness through clinical experiments (131, 132).

## Conclusions

Medicinal herbal plants and natural phytochemicals represent a powerful and valuable resource of active compounds that exhibit antiviral activities. Some of these sources require further chemical modification to enhance their potency and selectivity. Finding effective treatments for human and animal coronaviruses can benefit from testing several compounds and polyphenolics such as caffeic acid, isobavachalcone, myricetin, psoralidin, and quercetin, griffithsin, lycorine, silvestrol, and tryptanthrin. These compounds must be examined in *in vitro* and *in vivo* experiments to ensure their therapeutic and safe levels before conducting human clinical trials because of their toxicity at specific doses. The prime candidates for initial studies are these compounds that have been recognized by the FDA or other national or international organizations as generally safe or that have previously been allowed for medicinal uses. This review provides essential information on the potential role of phytochemicals in inhibiting coronavirus strains. This may provide adequate evidence to researchers seeking active therapeutic agents against SARS-CoV-2. Thus, naturally derived compounds alone or in integration with western medications may be further tested.

## Author Contributions

All authors listed have made a substantial, direct and intellectual contribution to the work, and approved it for publication.

## Conflict of Interest

The authors declare that the research was conducted in the absence of any commercial or financial relationships that could be construed as a potential conflict of interest.

## Footnotes

^1^https://www.worldometers.info/coronavirus/
